# High Body Mass Index Is an Indicator of Maternal Hypothyroidism, Hypothyroxinemia, and Thyroid-Peroxidase Antibody Positivity during Early Pregnancy

**DOI:** 10.1155/2015/351831

**Published:** 2015-07-27

**Authors:** Cheng Han, Chenyan Li, Jinyuan Mao, Weiwei Wang, Xiaochen Xie, Weiwei Zhou, Chenyang Li, Bin Xu, Lihua Bi, Tao Meng, Jianling Du, Shaowei Zhang, Zhengnan Gao, Xiaomei Zhang, Liu Yang, Chenling Fan, Weiping Teng, Zhongyan Shan

**Affiliations:** ^1^Department of Endocrinology and Metabolism, Institute of Endocrinology, Liaoning Provincial Key Laboratory of Endocrine Diseases, The First Affiliated Hospital of China Medical University, No. 155 Nanjing North Street, Shenyang, Liaoning 110001, China; ^2^Shenyang Women's and Children's Hospital, No. 87 Danan Street, Shenyang, Liaoning 110001, China; ^3^Department of Obstetrics and Gynecology, No. 202 Hospital of People's Liberation Army, No. 5 Guangrong Street, Shenyang, Liaoning 110001, China; ^4^Dalian Obstetrics and Gynecology Hospital, No. 1 Dunhuang Street, Dalian, Liaoning 116001, China; ^5^Department of Obstetrics and Gynecology, The First Hospital of China Medical University, No. 155 Nanjing North Street, Shenyang, Liaoning 110001, China; ^6^Department of Endocrinology, The First Affiliated Hospital of Dalian Medical University, No. 222 Zhongshan Road, Dalian, Liaoning 116001, China; ^7^Department of Endocrinology, No. 202 Hospital of People's Liberation Army, No. 5 Guangrong Street, Shenyang 110001, China; ^8^Department of Endocrinology, Dalian Municipal Central Hospital, Dalian Medical University, No. 826 Xinan Road, Dalian, Liaoning 116001, China; ^9^Department of Endocrinology, The First Hospital of Dandong, No. 76 Baoshan Street, Dandong, Liaoning 118001, China; ^10^Shenyang Women and Children Health Care Center, No. 74 Chongshan East Street, Shenyang, Liaoning 110001, China

## Abstract

*Background*. Maternal thyroid dysfunction in early pregnancy may increase the risk of adverse pregnancy complications and neurocognitive deficiencies in the developing fetus. Currently, some researchers demonstrated that body mass index (BMI) is associated with thyroid function in nonpregnant population. Hence, the American Thyroid Association recommended screening thyroid function in obese pregnant women; however, the evidence for this is weak. For this purpose, our study investigated the relationship between high BMI and thyroid functions during early pregnancy in Liaoning province, an iodine-sufficient region of China.* Methods*. Serum thyroid stimulating hormone (TSH), free thyroxine (FT4), thyroid-peroxidase antibody (TPOAb), thyroglobulin antibody (TgAb) concentration, urinary iodine concentration (UIC), and BMI were determined in 6303 pregnant women.* Results*. BMI ≥ 25 kg/m^2^ may act as an indicator of hypothyroxinemia and TPOAb positivity and BMI ≥ 30 kg/m^2^ was associated with increases in the odds of hypothyroidism, hypothyroxinemia, and TPOAb positivity. The prevalence of isolated hypothyroxinemia increased among pregnant women with BMI > 24 kg/m^2^.* Conclusions*. High BMI during early pregnancy may be an indicator of maternal thyroid dysfunction; for Asian women whose BMI > 24 kg/m^2^ and who are within 8 weeks of pregnancy, thyroid functions should be assessed especially.

## 1. Introduction

Thyroid dysfunction is the second most common endocrine disorder affecting women of reproductive age [1]. Abnormal maternal thyroid function is associated with various maternal and fetal complications such as preeclampsia, miscarriage, preterm delivery, and impaired neurodevelopment of the child [2–8]. To date, only few studies have explored the relationship between body mass index (BMI) and maternal thyroid dysfunction.

Autoimmune thyroiditis is believed to be the main cause of hypothyroidism in iodine-sufficient regions, and thyroid autoantibodies are believed to be the indicators of the disease [9], but only less than 33% of the patients with subclinical hypothyroidism were actually accompanied with positive thyroid-peroxidase antibody (TPOAb) [10]. Although iodine deficiency may lead to thyroid dysfunction, our previous study found that 1.9%–4.8% of the pregnant women with negative TPOAb are still subjected to subclinical hypothyroidism in iodine-sufficient region during their first trimester [11]. This fact indicates that thyroid autoimmunity and iodine status may not be the sole influencing factors of thyroid dysfunctions, suggesting the involvement of other contributing factors. Therefore, it is important to ascertain the influencing factor of maternal thyroid dysfunction for preventions and treatments of such diseases.

Obesity is a global epidemic and its prevalence has been increasing dramatically worldwide [12–15]. Many studies have suggested high BMI as an indicator of thyroid dysfunction among nonpregnant populations, and people with high BMI are more likely to have thyroid dysfunctions [16–18]. In 2011, the American Thyroid Association (ATA) guideline recommended a case-based manner for screening thyroid dysfunction and suggested “serum TSH testing should be carried out in pregnant women with morbid obesity,” but the scientific evidence for this is weak and particularly the relationship between BMI and maternal thyroid dysfunction during early pregnancy has not been well investigated [19, 20]. Hence, a large-scale population-based study focusing on “Subclinical Hypothyroid during Early Pregnancy (SHEP study)” in the cities of Liaoning province in China was conducted and 7953 pregnant women were enrolled. This is one of the preliminary reports of the SHEP study.

## 2. Subjects and Methods

### 2.1. Subjects

**Figure 1:**
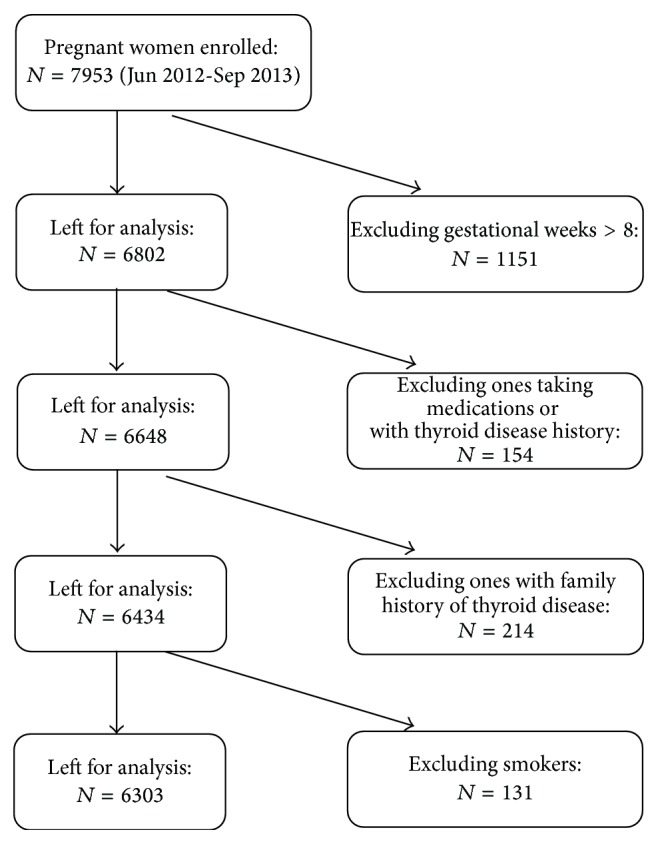
Flowchart of the study population.

The SHEP study was conducted in Dalian and Shenyang cities of Liaoning province, China. From June 2012 to September 2013, departments of obstetrics and gynecology and departments of endocrinology of nineteen hospitals had participated in this study. Recruitment criteria of this study included women aged 19–40 who have lived in the city for more than 10 years and planning to get pregnant or already with singleton pregnancy at the 4th–8th weeks of gestation. The following were excluded from this study: smokers; women who were >8 weeks pregnant; patients with a history of thyroid disease or any other chronic diseases; and patients on oral contraceptive regimens or any medical regimen that may affect thyroid function such as glucocorticoids, dopamine, or antiepileptic drugs. Of the 7953 pregnant women enrolled in SHEP study, 6303 pregnant women met the inclusion criteria of this study (Figure 1).

### 2.2. Methods

All subjects were asked to complete a questionnaire during their first hospital visit; subjects' serum TSH, FT4, TPOAb, and TgAb were measured, and their BMI and iodine concentration in urine were also determined. Samples of spot urine and blood were obtained from each participant in the morning after an overnight fast. All specimens were frozen at −20°C until analysis in one week. Serum TSH, FT4, TPOAb, and TgAb were measured in all participants using electrochemiluminescence immunoassay on Cobas Elecsys 601 (Roche Diagnostics, Switzerland). The functional sensitivity of serum TSH was 0.002 mIU/L. The intra-assay coefficients of variation (CV) of serum TSH, FT4, TPOAb, and TgAb were 1.57–4.12%, 2.24–6.33%, 2.42–5.63%, and 1.3–4.9%, respectively. The interassay CV values were 1.26–5.76%, 4.53–8.23%, 5.23–8.16%, and 2.1%–6.9%, respectively.

Urinary iodine concentration was determined in all participants by ammonium persulfate method based on Sandell-Kolthoff reaction. The intra- and interassay CV for urinary iodine concentration were 3-4% and 4–6% at 66 *μ*g/L and 2–5% and 3–6% at 230 *μ*g/L, respectively. Height and weight were measured with the participants wearing light clothes without shoes, height to the nearest 1.0 cm and weight to the nearest 0.5 kg. BMI was calculated as weight in kilograms divided by squared height in meters.

### 2.3. Diagnostic Standards for Thyroid Dysfunction

The Endocrine Society and American Thyroid Association guidelines recommended the usage of population-based trimester-specific reference ranges as diagnostic standard of thyroid dysfunction. Hence, the selected reference population was in accordance with Guideline 22 of the National Academy of Clinical Biochemistry [21]. As a result, the laboratory pregnant specific reference range of the 4th–8th gestational weeks was established [22]: TSH 0.29–5.22 mIU/L, FT4 12.27–20.72 pmol/L. The references of TPOAb and TgAb were provided by manufacturer: TPOAb 0–34 IU/mL and TgAb 0–115 IU/mL.

The following classifications were pregnancy-specific, and the reference values were created by our laboratory [22]:* overt hypothyroidism*: TSH > 5.22 mIU/L and FT4 < 12.27 pmol/L;* subclinical hypothyroidism*: TSH > 5.22 mUI/L with normal FT4;* isolated hypothyroxinemia*: FT4 < 12.27 pmol/L with normal TSH concentration;* TPOAb positivity*: TPOAb > 34 IU/mL;* TgAb positivity*: TgAb > 115 IU/mL.

### 2.4. Classification Criteria for BMI

According to the World Health Organization (WHO) criteria, BMI values can be divided into 4 classes: underweight (BMI < 18.5 kg/m^2^), normal weight (BMI between 18.5 and 24.9 kg/m^2^), overweight (BMI between 25.0 and 29.9 kg/m^2^), and obesity (BMI ≥ 30.0 kg/m^2^) [23].

### 2.5. Statistical Analysis

Kolmogorov-Smirnov method was used to test normality of the data distribution. Serum TSH and FT4 levels and urinary iodine concentrations failed the normality test; therefore, these variables were assessed using Kruskal-Wallis one-way analysis of variance on ranks in groups, and pairwise comparisons were performed using Mann-Whitney rank sum test. Pearson chi-square test was used to compare the prevalence rate of the disease.

Partial regression coefficients that express mean difference in FT4 per kg/m^2^ difference in BMI were estimated and tested whether this association displayed a linear trend (expressed as *P* values for trend). These assessments were done using both log-transformed and nontransformed BMI values, but the results were nearly identical, which led to the presence of nontransformed results.

In this study, bivariate analyses were in part exploratory; therefore, a *P* value of 0.008 (derived from the Bonferroni correction: 0.05/6 variables or groups of highly correlated variables) was deemed significant in these analyses. Multivariate analyses were rather confirmatory, and the conventional significance level of *P* < 0.05 was chosen to avoid type II statistical errors. All statistical analyses were performed with SPSS version 19.0 software.

### 2.6. Ethics Committee Approval

All research protocols were approved by the Medical Ethics Committee of China Medical University and were congruent with the Declaration of Helsinki. All mothers were provided with written informed consent after the research protocols were carefully explained to them.

## 3. Result

### 3.1. Iodine Status and Characteristics of the Study Population

According to historical data, Shenyang and Dalian are iodine-sufficient regions in China [24]. In this study, the median urine iodine concentrations (UICs) measured from 101 school children in Shenyang and 99 school children in Dalian were 191.2 *μ*g/L and 120.4 *μ*g/L, respectively. The median UICs of pregnant women in Shenyang and Dalian were 158.0 *μ*g/L and 152.8 *μ*g/L, respectively. Of the total 6303 pregnant women, 870 (13.8%) were underweight, 4547 (72.1%) were of normal weight, 796 (12.6%) were overweight, and 90 (1.4%) were obese.

### 3.2. Serum Levels of TSH and FT4 in Pregnant Women

**Figure 2:**
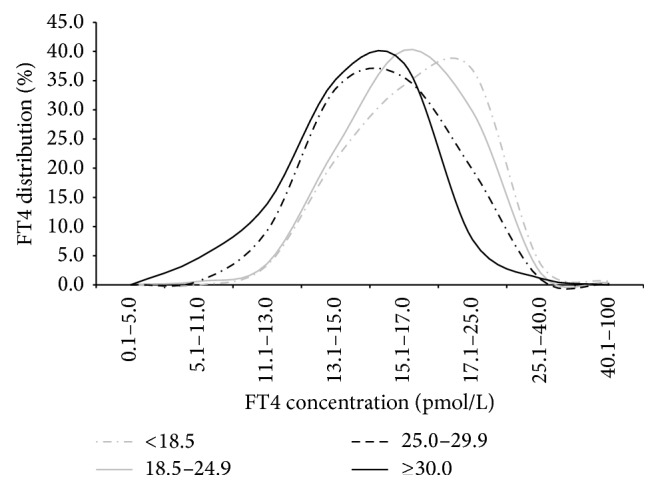
Distribution of FT4 in different group in pregnant women. In comparison to normal and underweight groups, obese and overweight groups resulted with left-shifted FT4 distribution curves; hence, the FT4 level was lower in groups with higher BMI.

Table 1 shows the median serum concentrations of TSH and FT4 in different BMI groups at the 4th–8th gestational weeks. TSH of normal weight and underweight groups did not exhibit any statistically significant differences. On the other hand, TSH was significantly higher in obese group than that in the overweight group (2.50 mIU/L versus 2.11 mIU/L, *P* < 0.008), and it was also higher in the overweight group than that in the normal group (2.11 mIU/L versus 1.86 mIU/L, *P* < 0.001). In contrast to the trend of TSH, the median concentration of FT4 decreased significantly as BMI value increased among all the groups. As a result, the distribution curve of FT4 in pregnant women was investigated (Figure 2). In comparison to normal and underweight groups, obese and overweight groups resulted with left-shifted FT4 distribution curves; hence, the FT4 level was lower in groups with higher BMI.

### 3.3. Prevalence of Thyroid Dysfunction

According to the pregnant specific reference ranges of the 4th–8th gestational weeks, the prevalence of thyroid dysfunction was obtained. As shown in Table 2, the prevalence of overt hypothyroidism, subclinical hypothyroidism, isolated hypothyroxinemia, TPOAb positivity, and TgAb positivity was 1.0%, 3.2%, 2.4%, 9.2%, and 12.5%, respectively, in pregnant women. The prevalence of overt hypothyroidism increased with the increase in BMI (*P* for trend <0.001). Although the prevalence of subclinical hypothyroidism had no statistical difference among four groups, the prevalence rate was highest in the obese group, reaching 7.8%. The prevalence of isolated hypothyroxinemia and TPOAb positivity increased with the increase in BMI (*P* for trend <0.001). Similar to TPOAb positivity, the prevalence of TgAb positivity increased with the increase in BMI (*P* for trend =0.004).

### 3.4. Multivariate Analyses

To assess the confounding factors and effect modifications, a multiple logistic regression analysis was applied. As shown in Table 3, four models were constructed. Model 1 evaluated the risk of elevated TSH (>5.22 mIU/L) in pregnant women, Model 2 evaluated the risk of reduced FT4 (<12.27 pmol/L), Model 3 evaluated the risk of TPOAb positivity (>34 IU/mL), and Model 4 evaluated the risk of TgAb positivity (>115 IU/mL). Results of Model 1 showed that obesity in pregnant women was associated with elevated TSH. Model 2 indicated that high BMI may act as a risk factor for hypothyroxinemia. Models 3 and 4 showed that high BMI may be an indicator of TPOAb positivity but not of TgAb positivity.

### 3.5. FT4 Variation with BMI and Cut-Off Value of BMI

**Figure 3:**
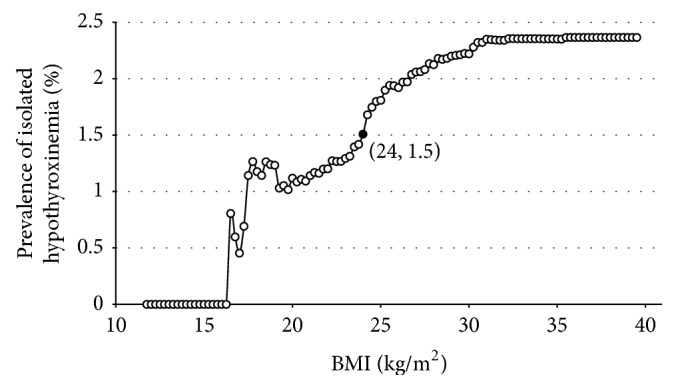
Prevalence of isolated hypothyroxinemia with the increase in BMI. The figure clearly indicates that the prevalence of isolated hypothyroxinemia during the 4th–8th gestational weeks shows a rise when BMI > 24 kg/m^2^.

For pregnant women who are in their 4th–8th weeks of gestation, FT4 was 0.12 pmol/L (95% CI, 0.10–0.17 pmol/L) lower for every 1 kg/m^2^ increment in the BMI (*P* < 0.05).  Figure 3 clearly indicated that the prevalence of isolated hypothyroxinemia during the 4th–8th gestational weeks showed a rise when BMI was >24 kg/m^2^.

## 4. Discussion

The present study indicates that high BMI may be an indicator of hypothyroidism, hypothyroxinemia, and TPOAb positivity during early pregnancy. To our knowledge, this is the first large-scale population-based study focusing on the relationship between maternal BMI and thyroid dysfunction during the 4th–8th gestational weeks in iodine-sufficient regions.

The present study showed that according to the pregnant specific reference ranges of the 4th–8th gestational weeks the prevalence of overt hypothyroidism and subclinical hypothyroidism was 1.0% and 3.2% in pregnant women. Multiple logistic regression indicated that BMI ≥ 30 kg/m^2^ was associated with elevated TSH. This finding is inconsistent with the previous studies done by Pop et al. and Gowachirapant et al. In Pop's study, a total of 1035 pregnant women including 470 overweight subjects failed to establish any correlation between BMI and TSH level during the 12th gestational weeks [25]. Similarly, Gowachirapant's study, which enrolled 131 overweight subjects with mean 11th gestational weeks, did not find that a high maternal BMI was a risk for elevated TSH in 514 pregnant women [26]. Additionally, with 9351 pregnant women at the 11th–20th gestational weeks, Haddow et al. were able to determine that no association is present between TSH value and body weight [27]. On the contrary, Bestwick et al. demonstrated that TSH was statistically influenced by weight in 21846 pregnant women during the 7th–16th gestational weeks [28]. The possible reason for the discrepancy of the lack of correlation between weight/BMI and TSH reported may be due to the differences in the number of subjects and the gestational weeks of subjects enrolled. It is known that human chorionic gonadotropin (hCG) synthesized by the placenta can stimulate thyroid hormone, causing a mild increase in FT4 concentration. A reciprocal decline in the serum TSH is most noticeable at the end of the first trimester, when the serum hCG concentrations peak [29]. Our study yielded a positive TSH value, possibly due to the fact that our study focused mainly on subjects at early gestational weeks, before serum hCG has reached a concentration sufficient enough to suppress TSH secretion. As for nonpregnant population, a systematic review done by de Moura Souza and Sichieri demonstrated that 18 of the 29 studies showed a positive relationship between measures of adiposity and serum TSH [30].

So, what may the underlying mechanism of the phenomenon that TSH is higher in the obese people mentioned above be? This may be that the adipose tissue is considered as an active endocrine organ that produces leptin, cytokine, and other inflammatory factors. Current researchers believe that leptin may play a key role in this process [16–18]. Both in vivo and in vitro experiments have shown that leptin may regulate the TRH and TSH via JAK-2 signal transducer and activator of transcription factor STAT-3 [31, 32]. So, it seems that leptin is linked to hypothalamic-pituitary thyroid axis via this mechanism.

The present study showed that FT4 was negatively correlated with BMI in the study population. Multiple logistic regression indicated that overweight and obese groups were associated with hypothyroxinemia, respectively. This was congruent with studies by Pop et al. and Gowachirapant et al., who also found a negative correlation between FT4 and BMI in pregnancy [25, 26]. In addition, studies done by Haddow et al. and Bestwick et al. also showed FT4 as negatively correlated with body weight during pregnancy [27, 28]. In nonpregnant population, Knudsen and Marwaha demonstrated that FT4 was negatively associated with BMI [33, 34]. Marzullo et al. also reported that FT4 and FT3 were relatively lower in obese subjects [35].

The present study showed that the prevalence of TPOAb positivity in overweight and obese groups was higher than that in the normal weight group. Also the prevalence of TgAb positivity in overweight and obese groups was higher than the prevalence found in normal weight groups. In order to control the confounding factors, a multiple regression was performed, which indicated that high BMI increased the risk of TPOAb positivity but not TgAb positivity. To the best of our knowledge, this is the first population-based study to show that maternal high BMI is correlated with TPOAb positivity. As for nonpregnant adults, there are some case control studies that indicated that the obese ones are more prone to become thyroid autoimmunity [17, 18, 35]. Marzullo et al. found a higher prevalence of hypothyroidism and TPOAb positivity in obese patients [35]. Although little research has been done during pregnancy, it has been found that obese people tend to have higher serum leptin concentration, and many studies have indicated that high leptin levels may be associated with autoimmunity [36]. Leptin is supposed to regulate the immune system by shifting the T helper balance toward a Th1 phenotype and suppress the function of T-regulatory (Treg) cells, which results in more TPOAb production [37, 38]. Furthermore, it is well known that autoimmune phenomena are suppressed during pregnancy. The present study focused on subjects at very early gestational weeks, hence leading to the finding of the association between high BMI and TPOAb positivity in pregnant women [39].

The clinical relevance of our study showed that Asian women who were within 8 weeks of pregancy, BMI > 24 kg/m^2^ might show a cut-off value for screening. This cut-off value we found was similar to the overweight standard given by WHO [23]. Furthermore, to correctly evaluate the thyroid function during pregnancy, overweight and obese individuals should be excluded from the reference population selected, while creating pregnancy-specific reference ranges.

There are two limitations in our study. Firstly, this study is a cross-sectional study, and a causal relationship between BMI and thyroid function has not been established. Large-scale and prospective epidemiological studies are needed for further confirmation. Secondly, leptin level was not determined in the current study; hence, the mechanisms of association between BMI and TSH levels and BMI and thyroid autoimmunity were obtained from literatures.

In summary, this study, which is the first of its kind that focused on women who were in the 4th–8th gestational weeks, has shown that high BMI is strongly correlated with hypothyroidism, hypothyroxinemia, and TPOAb positivity, but not TgAb positivity, during early pregnancy in an iodine-sufficient region. It is proposed for the first time that BMI > 24 kg/m^2^ may act as an indicator to screen thyroid function of Asian pregnant women in their early stage of pregnancy.

## Figures and Tables

**Table 1:** Serum levels of TSH and FT4 in pregnant women^a^.

BMI (Kg/m^2^)	TSH mIU/L			FT4 pmol/L		
	Median	2.5th–97.5th	*P* value	Median	2.5th–97.5th	*P* value
<18.5	1.85	0.17–5.50		16.52	12.95–22.18	
18.5–24.9	1.86	0.27–6.10	0.642	16.10	12.25–21.10	<0.001^b^
25.0–29.9	2.11	0.37–6.78	<0.001^b^	15.34	11.90–19.68	<0.001^b^
≥30.0	2.50	0.56–13.82	0.004^b^	14.67	10.25–18.77	<0.001^b^

TSH: thyroid stimulating hormone; FT4: free thyroxine.

^a^The *P* value represent the median level of this group compared with the upper group.

^b^ *P* < 0.008 was considered as a significant difference.

**Table 2:** Prevalence of thyroid dysfunctions^b,c^.

BMI (Kg/m^2^)	Total	Overt hypothyroidism (%)	Subclinical hypothyroidism (%)	Isolated hypothyroxinemia (%)	TPOAb positive (%)	TgAb positive (%)
Total	6303	63 (1.0)	203 (3.2)	149 (2.4)	578 (9.2)	790 (12.5)
<18.5	870	2 (0.2)	29 (3.3)	11 (1.3)	58 (6.7)	94 (10.8)
18.5–24.9	4547	42 (0.9)	141 (3.1)	87 (1.9)	402 (8.8)	564 (12.4)
25.0–29.9	796	16 (2.0)	26 (3.3)	40 (5.0)	97 (12.2)	114 (14.3)
≥30.0	90	3 (3.3)	7 (7.8)	11 (12.2)	21 (23.3)	18 (20.0)
*P* value		<0.001	0.101	<0.001	<0.001	0.025
*P*_*t*_ value^a^		<0.001	0.340	<0.001	<0.001	0.004

^a^ *P* value for trend.

^b^ *P* < 0.05 was considered as a significant difference.

^c^The diagnostic standards for thyroid abnormalities were according to the pregnant specific reference ranges of the 4th–8th gestational weeks.

**Table 3:** Multivariate logistic regression^a^.

BMI (kg/m^2^)	Adjusted OR (95% CI)			
	TSH > 5.22 mIU/L^b^	FT4 < 12.27 pmol/L^b^	TPOAb > 34 IU/mL^c^	TgAb > 115 IU/mL^d^
<18.5	0.90 (0.59–1.35)	0.56 (0.31–1.00)	0.80 (0.59–1.09)	0.88 (0.68–1.13)
18.5–24.9	Ref	Ref	Ref	Ref
25.0–29.9	1.33 (0.93–1.91)	2.43 (1.74–3.40)	1.53 (1.19–1.96)	1.02 (0.80–1.30)
≥30.0	2.41 (1.16–4.99)	5.34 (2.85–10.02)	3.18 (1.86–5.44)	0.89 (0.45–1.76)

Ref: reference category.

^a^Multivariate logistic regression was carried out in a stepwise manner. *P* < 0.05 was considered as a significant difference.

^b^Adjusted for age, gestational weeks, TPOAb, TgAb, and UIC (stepwise manner).

^c^Adjusted for age, gestational weeks, UIC, and TgAb (stepwise manner).

^d^Adjusted for age, gestational weeks, UIC, and TPOAb (enter manner).
